# Questions on Mediterranean Spotted Fever a Century after Its Discovery

**DOI:** 10.3201/eid1409.071133

**Published:** 2008-09

**Authors:** Clarisse Rovery, Philippe Brouqui, Didier Raoult

**Affiliations:** Unité des Rickettsies, Marseille, France

**Keywords:** Mediterranean spotted fever, *Rickettsia conorii*, synopsis

## Abstract

Mediterranean spotted fever (MSF) was first described in 1910. Twenty years later, it was recognized as a rickettsial disease transmitted by the brown dog tick. In contrast to Rocky Mountain spotted fever (RMSF), MSF was thought to be a benign disease; however, the first severe case that resulted in death was reported in France in the 1980s. We have noted important changes in the epidemiology of MSF in the last 10 years, with emergence and reemergence of MSF in several countries. Advanced molecular tools have allowed *Rickettsia conorii conorii* to be classified as a subspecies of *R. conorii*. New clinical features, such as multiple eschars, have been recently reported. Moreover, MSF has become more severe than RMSF; the mortality rate was as high as 32% in Portugal in 1997. Whether *Rhipicephalus sanguineus* is the only vector and reservoir for *R. conorii conorii* is a question not yet answered.

## Abstract 2

New findings in MSF epidemiology, clinical features, and severe forms have changed the general perception of MSF.

Mediterranean spotted fever (MSF) is a tick-borne disease caused by *Rickettsia conorii*. It was first described a century ago as a disease that caused high fever and spots (*1*). Our knowledge about MSF has evolved since its first description. First, we thought that MSF was only limited to some regions of the world, i.e., southern Europe, North Africa, and India. In fact, an increasing number of regions have been reporting MSF cases, such as central Europe and central and southern Africa. Serologic techniques cannot distinguish among different rickettsiae species of the spotted group. Consequently, all rickettsioses with spotted fever group (SFG) antibodies were considered to have MSF in countries where this disease was endemic. Early clinical descriptions that relied only on serologic test results were likely to include infections related to multiple rickettsial species and were probably not describing a unique entity. For example, in France, emerging rickettsioses caused by bacteria, including *R. sibirica mongolitimonae, R. slovaca, R. felis, R. helvetica,* and *R. massiliae,* have been recently described (*1*). The first description of patients with MSF in southern France may have included patients with these emerging rickettsioses. With new molecular tools such as PCR and sequencing, we can now identify much more precisely the rickettsial agent responsible for the disease.

MSF is an emerging or a reemerging disease in some countries. For example, in Oran, Algeria, the first case of MSF was clinically diagnosed in 1993. Since that time, the number of cases has steadily increased (*2*). In some other countries of the Mediterranean basin, such as Italy and Portugal, incidence of MSF has substantially increased in the past 10 years.

Another point is that MSF was considered for 70 years a benign disease when compared with Rocky Mountain spotted fever (RMSF). In fact, because of the lack of medical interest in MSF, its real severity was long ignored. Although the mortality rate was evaluated to be from 1% to 3% in the early reports in the literature, the first description of a highly severe form of MSF was published in the early 1980s (*3*). At present, we know that MSF is at least as severe as RMSF and has a mortality rate as high as 32.3%, which occurred in Portugal in 1997 (*4*).

Although many hypotheses have been suggested, the nature and distribution of the reservoir of the rickettsiae in nature are still not answered. The aim of this review is to show the evolution in our knowledge of MSF in the past 10 years with an emphasis on epidemiology, clinical features, and severe forms.

## Historical Background

The historical background of MSF is summarized in Table 1. MSF was described in Tunisia by Conor and Bruch (*1*) and was soon reported in other regions around the Mediterranean basin. The disease was thereafter also known as boutonneuse fever (spotted fever) because of the manifestation of a papular rather than a macular rash. The typical inoculation eschar, the *tâche noire* (black spot), was described in 1925 in Marseille by Boinet and Pieri (*5*). In the early 1930s, Durand and Conseil (*6*) proposed that the brown dog tick, *Rhipicephalus sanguineus,* was the vector in Europe after they inoculated humans with crushed ticks. Blanc and Caminopetros successfully repeated these experiments on humans and spermophiles (*7*). Brumpt showed that the SFG rickettsia was the causative agent, and in honor of Conor, this organism was named *R. conorii.* Blanc and Caminopetros showed that *R. conorii* could be transmitted through transovarial passage (*7*) in ticks and hypothesized that ticks could be the reservoir of *R. conorii* (Table 1).

**Table 1:** Historical reports of MSF*

Year	Discovery	Authors†
1910	Description of the “fièvre boutonneuse de Tunisie” (7 cases)	Conor and Bruch
1925	Description of a cluster of MSF (8 cases) in Marseille, France, during the summer	Olmer
1927	Description of the inoculation eschar, the *tache noire* (black spot). Description of the disease associating fever, spots, and eschar as “Marseille fever”	Boinet and Pieri
1930	Experimental transmission of the disease by the brown dog tick	Durand and Conseil
1932	Demonstration of the transstadial and transovarian transmission of the agent of MSF in ticks. Demonstration of *Rickettsia* in infected ticks	Blanc and Caminopetros
1932	Isolation of the *Rickettsia* causing MSF in the vagina of infected guinea pigs and in infected ticks; named *R. conorii*	Brumpt
1982	First description of cases of malignant MSF	Raoult

*MSF, Mediterranean spotted fever. †First or senior authors.

## Knowledge Gained about MSF in the Past 10 Years

### New Information about the Agent

**Figure 1:**
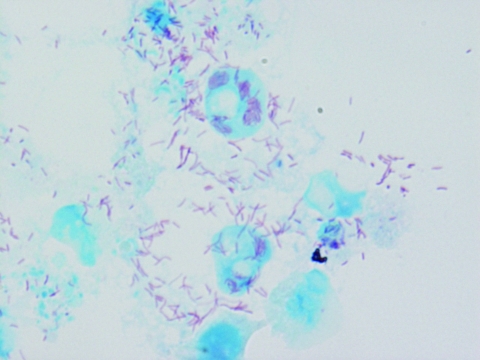
*Rickettsia conorii conorii* observed in Vero cells (red rods; magnification ×1,000).

**Figure 2:**
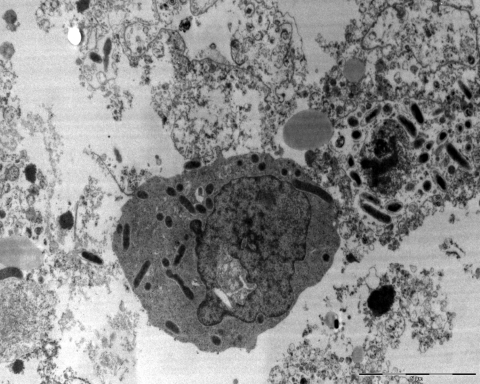
*Rickettsia conorii conorii* localized in cytoplasm of host cells as seen by electron microscopy (magnification ×100,000).

*R. conorii* is an obligate, intracellular, gram-negative bacterium (Figures 1, 2). In recent years, the rickettsial field has undergone a substantial evolution, particularly because of the technologic advances in molecular genetics. In the past decade, several rickettsial genomes, including that of *R. conorii* (*8*), have been sequenced. Availability of these genomic data have allowed, in turn, the development of global approaches, including proteomics and transcriptomics, powerful tools to gain a better knowledge of cell biology and interaction of rickettsiae with their host cells. Due to genome sequencing, the taxonomy of rickettsiae has undergone extensive reorganization. Until 2005, opinions were divided as to whether rickettsial strains related to *R. conorii* belonged to the same species or were distinct species. This included Israeli spotted fever rickettsia, Indian *R. conorii* strain (Indian tick typhus rickettsia [ITTR]), and Astrakhan spotted fever rickettsia (AFR) (Table 2). In fact, phylogenetically, these rickettsiae and *R. conorii* strain Malish (the agent of MSF) constitute a homogeneous cluster supported by significant bootstrap values and distinct from other *Rickettsia* spp. By estimating the degrees of genotypic variation among isolates of the *R. conorii* strains Malish, ISFR, ITTR, and AFR, Zhu et al. proposed that *R. conorii* species nomenclature should be modified through the creation of the following subspecies: *R. conorii conorii*, *R. conorii caspia*, *R. conorii* *israelensis*, and *R. conorii indica* (*9*). These rickettsiae have discernable serotypes and cause diseases with distinct clinical features in defined geographic locations, but they are not genetically different enough to be considered as new species.

**Table 2:** Distribution, vector, and main clinical features of the different subspecies of *Rickettsia conorii* complex

Rickettsia	Vector tick	Geographic repartition	Human disease name	Symptoms present, % patients			Fatal forms? (% patients)
				Fever	Inoculation eschar	Rash	
*R. conorii conorii*, isolates Malish, Moroccan Kenyan	*Rhipicephalus* sp., *Haemaphysalis leachii*	Mediterranean area (southern Europe, northern Africa), Croatia, Slovenia, Kenya, Somalia, South Africa, and surrounding the Black Sea (Turkey, Bulgaria, Ukraine, Romania)	Mediterranean spotted fever	91–100	20–87	93–100	Yes (0–18.1)
*R. conorii israelensis*	*Rh. sanguineus*	Israel, Portugal, Sicily	Israeli spotted fever	100	0–46	98–100	Yes (0–3.5)
*R. conorii caspia*	*Rh. sanguineus, R. pumilio*	Astrakhan region, Chad, Kosovo	Astrakhan spotted fever	100	23	94	No
*R. conorii indica*	*Rh. sanguineus*, *Boophilus microplus*, *H. leachii*	India, Pakistan	Indian tick typhus	100	Rare	100 (frequently purpuric)	No

### New Information about Epidemiologic Features

MSF is endemic to the Mediterranean area, including northern Africa and southern Europe. Cases are still identified in new locations within this region. Thus, some cases were recently described in Algeria, Malta, Cyprus, Slovenia, Croatia, Kenya, Somalia, South Africa, and in areas surrounding the Black Sea (Turkey, Bulgaria, and Ukraine). Spotted fever cases have been confirmed as MSF by the use of molecular tools in Portugal, Italy, Malta, Greece, Croatia, Spain, France, Turkey, Algeria, Tunisia, Morocco, Zimbabwe, Kenya, and South Africa. MSF is suspected to be endemic in Slovenia, Albania, Ukraine, Georgia, and Zimbabwe, but *R. conorii conorii* has not been isolated in human clinical samples in these countries.

**Figure 3:**
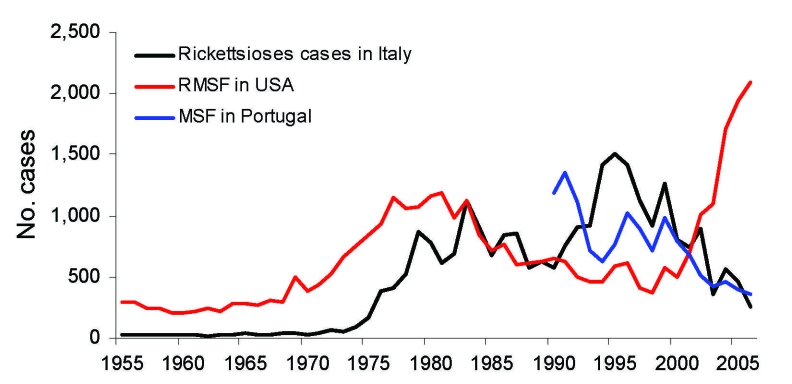
Fluctuation of incidence of Mediterranean spotted fever (MSF) in Italy and Portugal and of Rocky Mounted spotted fever (RMSF) in the United States, by year.

**Figure 4:**
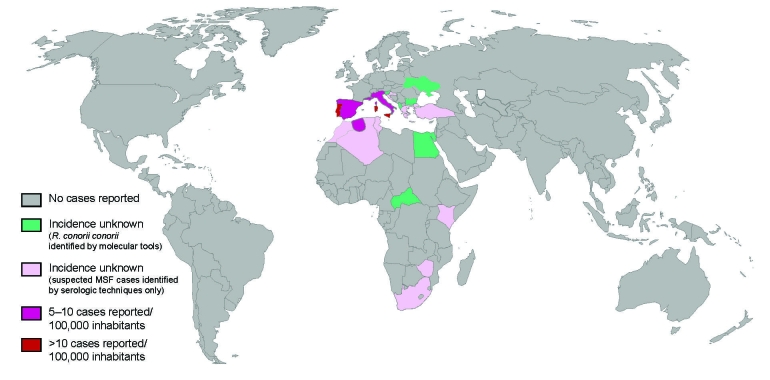
Distribution of the cases of Mediterranean spotted fever (MSF) in the world and incidence of the disease in countries where MSF is endemic.

MSF appears to be waxing and waning, as indicated by peaks in the number of MSF cases (Figure 3). Incidence of the disease sharply increased in the 1980s in Italy (*10*), Spain, and southern France (*11*). In some countries, MSF is reemerging. During the past decade in Portugal, the number of hospitalizations has increased from <200 to >400 cases per year (*4*). In Italy, a notable increase in case numbers was reported during the 1990s; cases peaked in 1999 at 699. In Bulgaria, MSF cases started to sharply decrease at the beginning of the 1960s and completely disappeared in the 1970s. However, in 1995, a peak of MSF disease with 236 cases was noticed in this country and reached 716 cases in 1997 (*12*). In Oran, Algeria, the first case of MSF was diagnosed in 1993; since that time, the number of cases has steadily increased to reach 134 in 2004 (*2*). Figure 4 illustrates the distribution and incidence of *R. conorii conorii* infection in countries where MSF is endemic.

Such variations have also been noted for RMSF (*13*). In the United States, the disease is in the midst of its third emergence since 1920, after peaking from 1939 to 1949, and again from 1974 to 1984 (*13*,*14*) (Figure 3). The causes of these variations in the incidence of MSF and other SFG rickettsioses are unknown. In most countries, no national epidemiologic surveillance of MSF cases is conducted. Only Italy and Portugal have a formal surveillance program. However, in these countries, surveillance is passive and not mandatory. Thus, in many countries, reported cases depend on the observers and can be affected by such variables as the need for international publications by physicians. For example, the dramatic increase in MSF cases in Oran, Algeria, is mainly due to the renewed interest in the disease by 1 physician (*2*). However, in countries that have a surveillance program, incidence of MSF cases actually varies in time. Another factor that limits the study of incidence of MSF in European countries is that nonspecific serologic tests are used for the diagnosis of MSF and could include other SFG rickettsioses. However, in Orán, when specific tests are used such as Western blot and molecular tools, *R. conorii conorii* appears to be the main etiologic agent of SFG rickettsioses in this area (D. Raoult, unpub. data). An increased number of ticks and increased human contact with the habitat of infected ticks are possible factors that would explain variations of incidence. In addition, the ecologic changes in the outskirts of large cities during the 1980s may have played an important role by moving rural sources to suburban zones. Climatic factors could also intervene, such as the increase of temperature, the lack of rainfall (for example, in Spain [*15*]), or the reduced number of days of frost during the past year in France (*16*). Climatic variations are suspected to play an important role in tick activity and, consequently, on rickettsial prevalence (*15*). It is also conceivable that undetected reservoir–vector systems have emerged or that the size of the reservoir has increased. This increase in MSF cases in the 1970s could also have been caused by a shift in effectiveness of prescribed antimicrobial drugs. In fact, before the 1970s, doxycline was used as an empiric therapy for patients with a fever of unknown origin. Finally, dramatic increases in MSF cases during the 1970s may be related to the advent of new diagnostic methods, such as microimmunofluorescence and the increased interest in traveling to several countries such as France, Italy, and Spain. Sporadic cases in non–disease-endemic countries are also observed as a consequence of tourism (*17*).

### New Information about Clinical Features

**Figure 5:**
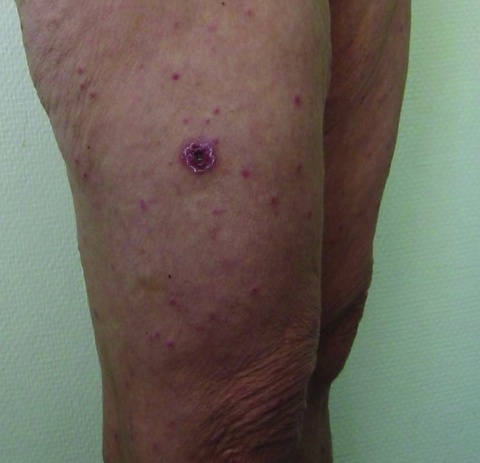
Typical eschar and spots on the leg of a patient with Mediterranean spotted fever.

The clinical description of MSF has not really changed since its it was first described. MSF is characterized, just as the other rickettsioses, by fever, headaches, and maculopapular rash. Most of the studies reporting series of patients with MSF could have been affected by many factors. Descriptions of the first clinical cases, which were diagnosed on the basis of serologic test results alone, surely included infections related to other species of rickettsiae. Clinical descriptions with a series of patients are also subject to biased observations. For example, the eschar (Figure 5) can be difficult to retrieve and can sometimes be atypical, for example, having the aspect of a furuncle, which is difficult to recognize. This could explain wide variations in the reported presence of an eschar (20%–86%) (*4*,*11*).

Eschars are rarely multiple. This observation was, however, reported in the early description of the disease by Olmer (*5*). More recently, 2 studies in Sicily found multiple eschars in 7 (1.08%) of 645 children (*18*) and in 6 (1.4%) of 415 children (*19*), respectively. In studies in Spain (*20*,*21*), multiple eschars were found in 3%–11.5% of patients and involved more children. These findings should, however, be interpreted with care. The role of *R. aeschlimannii* circulating in *Hyalomma* spp. in Spain has to be considered in the cases of multiple eschars. Indeed, *Rh. sanguineus* has a low probability of biting humans, and the infection rate by rickettsiae is low (<10%). Accordingly, the probability of being bitten simultaneously by several infected *Rh. sanguineus* is low. Conversely, *H. marginatum* ticks readily bite humans, and persons may receive multiple simultaneous tick bites (*1*). Moreover, in Spain, Fernandez-Soto et al. reported *R. aeschlimannii* in 6 species that frequently feed on humans; a total of 4,049 ticks were removed from 3,685 asymptomatic patients. In this study, *R. conorii conorii* was isolated from only 1 *Rh. sanguineus* (*22*). In this context, we can hypothesize that cases of spotted fever acquired in southern Europe and associated with the presence of several eschars can be caused by *R. aeschlimannii.* Nevertheless, multiple eschars also exist in MSF. In 2004, our laboratory confirmed a diagnosis of MSF in 9 patients by using PCR. Among them, 3 had multiple eschars, and 2 of the 3 had a severe form of MSF (D. Raoult, unpub. data). All of these patients were bitten in the southern of France. In Algeria, Mouffok et al. reported in a prospective study of 20 of 270 patients with multiple eschars (D. Raoult, unpub. data).

### New Information about the Severity of MSF

#### Historical Background

Although mortality rates were determined to be 1%–3% in the early description, before the antimicrobial drug era, MSF was thought to be a benign illness with the proportion of deaths <1%. MSF was even named benign summer typhus. In comparison, RMSF was described as a very severe disease with a mortality rate of 65%–90% in western Montana (*23*). However, a few complications have been reported, including renal, neurologic, cardiac, phlebitis, and retinal complications. Severe forms of MSF were reported in 1981 (*3*); 4 of 6 patients with confirmed diagnoses died. Active surveillance of MSF in Marseille and the surrounding area in 1983–1984 found 7 (5%) of 142 patients with a severe form of MSF. At the same time, severe cases of MSF were described in Spain (*24*). Severe cases of MSF were similar to severe cases of RMSF with purpuric rash associated with neurologic manifestations and multiorgan dysfunction syndrome. At this time, *R. conorii conorii* was the sole agent in cases of spotted fever known in these countries. However, we could not exclude the possibility that some of these severe cases could be caused by rickettsiae discovered since this time. In fact, in 1997, in Portugal, some of the severe cases were caused by *R. conorii israelensis* (*4*). Recently, a severe spotted fever case caused by *R. australis* has been reported (*25*); the organism was previously considered to be responsible for a benign disease.

#### Temporal and Geographic Distribution of Severe Cases

Severity of MSF varies according to the time. For example, in 1983, in Salamanca, Spain, MSF was reported as a severe disease with complications occurring in 19% of the cases (*24*). During the 2 preceding years, in the same area, the annual incidence of complications was 3.7% (1981) and 4.34% (1982) (*24*). In 1997 in Beja, a southern Portuguese district, the mortality rate in hospitalized patients with MSF was 32.3%, the highest obtained there since 1994 (*4*). The mortality rate for the previous years was <15% in this region. This example illustrates not only a temporal, but also a geographic variation in the severity of MSF. Geographic variation in severity has also been reported for RMSF. In fact, in the United States, the overall case-fatality rate was 1.4% during 1997–2002 (*14*); however, in Brazil, the average case-fatality rate during 1995–2004 was 29.1% (*13*).

Recent MSF appears to be a more severe disease than it was in the past. Mortality rates were 3.2% in Oran, Algeria, in 2004 (*2*); 5.6% in Marseille, France, in 2003 (D. Raoult, unpub. data); and 32.3% in hospitalized patients in Beja, Portugal, in 1997 (*4*). This increase in severity has not been explained. Indeed, MSF is more quickly recognized and treated with more effective antimicrobial drugs than in the past. Thus, we would expect that MSF would be less severe. This was the case for RMSF, which had mortality rates in the United States of 2.4% during 1993–1996 (*26*) and 1.4% during 1997–2002 (*14*), in contrast to a mortality rate of 65%–90% at the beginning of the century. The first hypothesis could be that severe forms of MSF were not well recognized in the past. In fact, MSF was considered to be a benign disease before the 1980s (*27*). MSF was not a diagnosis that was evoked when patients were hospitalized in intensive care with a febrile rash. Another hypothesis is that more virulent strains of *R. conorii conorii* appeared. At present, however, the use of highly variable intergenic spacer sequences for multispacer typing of *R. conorii conorii* strains has not led to the identification of a more virulent strain (*28*). Remarkably, MSF is not a severe disease in children. No deaths or severe cases were noticed in 60 children in Barcelona during 1979–1980 (*29*). In Sicily, no severe forms were reported in 645 children during 1984–1996 (*18*) or in 415 children during 1997–2004 (*19*). Only 1 report of death (in a 16-year-old patient) was found in the literature (*30*). However, these studies could have been affected by recruitment bias. Other risk factors for severe MSF, other than advanced age, include the following: immunocompromized situations, chronic alcoholism, glucose-6-phosphate dehydrogenase deficiency, prior prescription of an inappropriate antimicrobial drug, delay in treatment (*1*), and diabetes (*4*).

## Unanswered Questions

### Does *R. conorii conorii* Have Vectors Other than *Rh. sanguineus*?

**Figure 6:**
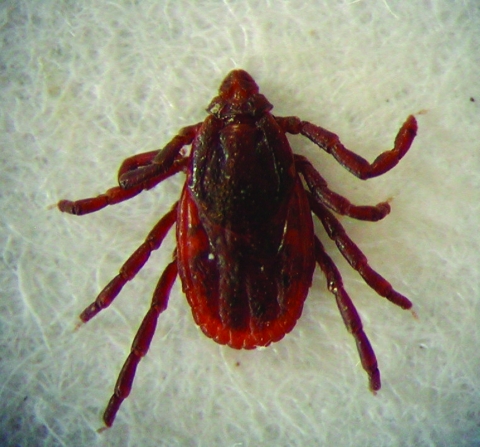
*Rhipicephalus sanguineus* adult tick, the suspected vector for *Rickettsia conorii conorii.*

*Rh. sanguineus* (Figure 6) is generally accepted as the main vector for *R. conorii conorii* in Europe and North Africa. No other species of ticks have been retrieved from the skin of humans infected with *R. conorii conorii*. *Rh. sanguineus* has a weak affinity for humans, as evidenced by the fact that only 3.5% of larvae, 2% of nymphs, and 5% of adults settle on humans when placed in direct contact with them (*31*). However, according to a 2003 report, 22 *Rh. sanguineus* (1 adult and 21 nymphs) were found attached to a homeless man with alcoholism, who was living with his dog near Marseille (*32*). Because this infestation was associated with the highest summer temperature noted in France in the past 50 years, the host-seeking and feeding behavior of *Rh. sanguineus* ticks may have been altered by the unusual climatic circumstances. Multiple eschars indicate that the same tick has bitten patients several times or that multiple ticks have bitten the patient. In southern France, we never noticed multiple eschars until 2004, when 2 patients had 3 eschars and 1 patient had 2 eschars (D. Raoult, unpub. data). Patients with multiple eschars were not observed in 2005. Multiple eschars could indicate recent modification of tick behavior related to unusual climatic circumstances of the previous year. Likewise, laboratory evidence has shown an association between changing temperature and changing behavior of *Rh. sanguineus* (D. Raoult, unpub. data).

In Africa, vectors other than *Rh. sanguineus* could intervene. PCR, followed by restriction fragment length polymorphism, on samples of hemolymph-positive ticks in Zimbabwe showed *R. conorii conorii* to be present in *Rh. simus* and *Haemaphysalis leachi* (*33*). In our laboratory, we recently isolated *R. conorii conorii* from *H. punctaleachi* collected in Uganda (D. Raoult, unpub. data).

### What Is the Real Reservoir of *R. conorii conorii?*

Because of transovarial transmission, *Rh. sanguineus* was thought to be the reservoir for *R. conorii conorii* (*7*). However, this commonly accepted idea is now being challenged. In fact, only a small proportion of *Rh. sanguineus* ticks are infected with *R. conorii conorii*; infection rates are generally <15% (*34*). Moreover, most *Rh. sanguineus* ticks experimentally infected with *R. conorii conorii* die (*35*). This increased proportion of deaths in *Rh. sanguineus* has also been shown for naturally infected ticks after 1 generation (P. Parola, unpub. data). Curiously, *Rh. sanguineus* is found throughout the world, but *R. conorii conorii* is found only in some regions of the world. Dogs, the usual hosts of *Rh. sanguineus,* are also found everywhere. Even within endemic zones, microfoci exist. Early rickettsiologists such as Olmer in southern France and Blanc and Caminopetros in Greece have shown that foci of MSF are usually small with a low propensity for diffusion (*7*). Clusters in very limited geographic zones have also been observed for *Rh. sanguineus* that transmit *R. rickettsii* infection in Arizona (*36*). One explanation might be that transovarial transmission may occur for a limited number of passages and that *Rh. sanguineus* may only be the vector of the disease.

Currently, we do not know the real reservoir for *R. conorii conorii*. Dogs serve as common transport hosts by bringing infected ticks closer to their owners. In certain zones of southern Europe, a correlation between the percentage of the canine population with antibodies to *R. conorii conorii* and the incidence of MSF in humans has been found (*37*). Seropositivity was even higher in dogs belonging to MSF patients (*37*). Dogs are transient reservoirs because of transient rickettsemia after infection; therefore, dogs do not seem to be an efficient reservoir for *R. conorii conorii*. Evidence has recently been shown that dogs can exhibit febrile illness related to infection with this bacterium (*38*). In the early description of MSF, Pieri showed that rabbits could be bacteremic without severe disease developing, which suggests that these animals could be a reservoir for *R. conorii conorii*. Le Gac et al. suggested that wild rabbits (*Oryctolagus cuniculus*) could play a role in the transmission of *R. conorii conorii* on the French Mediterranean coast because a large drop in MSF cases occurred in 1952 during an outbreak of myxomatosis, which killed all the wild rabbits on the French Mediterranean coast. MSF reappeared in 1967 with the reappearance of wild rabbits (*39*). Ruiz Beltran et al. (*40*) found that 76.5% of wild rabbits and 25% of hares had antibodies to *R. conorii conorii* in Salamanca, Spain. Hedgehogs and other small rodents are also candidates for the reservoir because antibodies against rickettsiae have been detected in serum of these animals (*39*). Because *R. conorii conorii* has never been isolated in the Americas, its reservoir is most likely a mammal present only in the Old World that has yet to be determined.

## Conclusion

Our knowledge regarding MSF has undergone notable changes within the past 10 years. Molecular tools have allowed us to better discriminate rickettsial species and subspecies of the SFG. We now know that >1 rickettsiosis can be present in the same country. Patients who have been included in series of MSF cases may have had other rickettsioses. Moreover, MSF has a wider distribution than previously described. The disease has emerged and reemerged in several countries in the Mediterranean basin. New clinical features, such as multiple eschars, previously suggested in the early description, have now been confirmed in MSF. MSF is becoming an increasingly severe disease with death rates ranging from 3.2% to 32%. However, questions persist regarding the vector and reservoir for this disease, which need to be addressed.
